# "I have a personal claim to myself": a visually impaired student's perspective on her participation in physical activity and physical education settings

**DOI:** 10.3389/fspor.2025.1585254

**Published:** 2025-05-15

**Authors:** Anne Bödicker, Sandra Elisath

**Affiliations:** ^1^ University of Education Karlsruhe, Karlsruhe, Germany; ^2^ University of Paderborn, Paderborn, Germany

**Keywords:** inclusion, narrative interview, sports, teacher—education, vulnerability

## Abstract

In our daily lives, we often come across ideas and approaches that are intended to support and enhance our well-being, with the aim of achieving positive results, such as resilience and health, if implemented effectively. This trend can be understood as part of the long history of disciplining and normalizing the body in Western societies. Functioning, keeping up, and being resilient in our fast-paced society now appear to be the social norm. This status quo creates a link to physical education (PE) as a body-related school subject, where the focus is on the body and its performance. Several recent studies have explored the experiences of people with visual impairment (VI) in PE. For young people with blindness and visual impairment (BVI), sports and physical activity (PA) are deemed beneficial as they promote physical and mental health while increasing well-being and life satisfaction. These factors—well-being and life satisfaction—are closely intertwined with the concept of resilience. However, resilience cannot be conceived without acknowledging vulnerability, which people embody to different degrees. Vulnerability represents a human condition, as all people are potentially vulnerable. What can this concept mean for adolescents who are assigned to a so-called vulnerable group? This article aims to explore this question from a biography-oriented perspective. By adopting a critical perspective of Ableism within the context of Disability Studies, we reclassified societal attributions of abilities. This article focuses on a semi-narrative guided interview conducted with a 15-year-old teenager with BVI who had recently transitioned from inclusive mainstream schools to a state-approved special school for the visually impaired. Thus, she has had diverse experiences in both segregated and inclusive educational settings. This study highlights the extent to which empowering personal and non-personal factors are perceived as such and examines their interaction with participation in sports contexts. The findings critically reflect on the teachers' role in either enabling or hindering participation in PE, emphasizing the need for inclusion-sensitive approaches in teacher education.

## 1 Introduction

In our daily lives, we often come across ideas and approaches that are intended to support and enhance our well-being, with the aim of achieving positive results, such as resilience and health, if implemented effectively. This “trend” can be understood as an additional step in the long history of disciplining and normalizing the body in the West, referred to by Brinkmann in 2018 (1). Functioning, keeping up, and being resilient in our fast-paced society now appear to be the social norm or idea of reality (2). This status quo creates a link to physical education (PE) as a body-related school subject [“Körperfach” (3)], where the focus is on the body and its performance (4, 5).

Several recent studies have explored the experiences of people with visual impairment (VI) in PE (6–9). For young people with blindness and visual impairment (BVI), sports and physical activity (PA) are deemed beneficial, as they promote physical and mental health along with improved well-being and life satisfaction (10).

These factors—well-being and life satisfaction—are closely intertwined with the concept of resilience (11). However, resilience can be better understood after first acknowledging the concept of vulnerability, which people embody to different degrees. Hirschberg and Valentin (12), for example, spoke of vulnerability as a human condition, as all people are potentially vulnerable. According to Klein (13), it appears to be particularly challenging to recognize and accept one's own vulnerability in performance-oriented societies. On the contrary, the fact that vulnerability and dependency are indispensable parts of human existence is often rejected, avoided, and considered taboo (13). Contrary to the authors who focus on Disability Studies, social or (educational) political perspectives on disability attribute a vulnerability to certain groups, thereby singling them out as individuals or groups. What can this concept mean for adolescents who are assigned to a so-called vulnerable group? In this article, we explored this question in a biography-oriented way. As part of the evaluations that are typically conducted in Disability Studies, we adopted a critical perspective of ableism, that is, we critically classified attributions of abilities (14). In this article, we focused on a semi-narrative guided interview with a 15-year-old teenager with BVI named Kai. Kai had been attending a state-approved special school for the visually impaired at the time of the interview for only 3 months, after attending an inclusive working regular school for over 9 years. Thus, she had different experiences from attending both segregated and inclusive schools.

We demonstrated the extent to which personal and non-personal factors that are generally described as empowering [e.g., positive experiences of self-efficacy, self-perception, the belief that one is able to influence one's own life, social skills, understanding and recognition by adults or friendly relationships with peers, cf. (15, 16)] are also perceived as such. We achieved this aim by examining the ways in which different forms of participation and stress appear in the context of sport. In the following section, we provide a brief overview of the general research project (Section 2)^1^ before we analyze the interview excerpts (Section 3). In the final step in the process, we discuss how the forms of (pedagogical) support described above are critically categorized in the context of reflexive, inclusion-sensitive teacher education (Section 4). We pay particular attention to the importance of teachers' emotions or affective resonance [in the context of disability (18)], which can be partly responsible for participation or barriers in PE, and which need to be recognized.

## 2 Methods/analysis

As part of the project [“Jugend—Schule—Dis/ability: Rekonstruktion institutioneller, peer- und familiärer Erfahrungen von Förderschüler*innen mit Sehbeeinträchtigung”, (17)], eight semi-narrative guided interviews were conducted with young people between the ages of 15 and 19 who were attending a state-approved special school for the visually impaired at the time of the interview. The unique feature of this school was that all standard German school-leaving qualifications up to the highest German school diploma could be completed there. From the perspective of regular school careers, it should be noted that all these young people had repeated at least one school year and had changed schools (and school types) frequently (19),^2^ although they had different previous experiences in both segregated and inclusive schools. All participants and their parents gave informed consent, and pseudonyms were used to protect participants' privacy.

Although the topic of sport was not the primary focus of the study, this topic was explicitly addressed by one person. For this reason, we focused on Kai's perspective in more detail, as the importance of sport and PE was a central topic for her in the context of her identity-related discussions.

### 2.1 Methods

On the basis of a culturally construed understanding of disability, we claim that the difference between blind and partially sighted is irrelevant in this study. Kai had been diagnosed as blind. However, the results showed that no matter which diagnosis was applied to the students, once they were ascribed as students with VI, all of them had to cope with disability ascriptions.^3^

As part of evaluations within Disability Studies, we adopted a critical perspective of ableism (i.e., attributions of abilities are critically classified). This perspective is based on Campbell's (14) understanding of ableism as a network of beliefs, processes, and practices, which create a particular kind of self and body as perfect. From this understanding, disability is seen as an inferior state of being human. Zinsmeister specified: “In terms of the ableist norm, normal or natural means that people can see, hear and communicate verbally and in writing without restriction, that they are mobile without restriction and as productive as possible.” Linked to this definition are expectations of a certain social behavior, an outward appearance, and a general ability to function (20).

Hirschberg and Köbsell (21) noted that, within this paradigm, the cultural depth of ableist thinking is inextricably intertwined with the fabric of social dominance. The pervasiveness of this cognitive framework, which is ingrained through socialization, makes it a subject that is rarely called into question. Adopting a critical research perspective enables the examination and interrogation of social and institutional norms, normalities, values, and (il)legitimate attributions of ability, as well as traditional representations of disability. This critical scrutiny is particularly pertinent in the context of PE teachers in schools, where (a lack of) ability can become particularly evident during PE. The significance of PE in this context is underscored by its “elementary reference to the body,” which enables “diversity to be experienced here in the flesh” (5). To become aware of their particular significance and scope as teachers in this field, it is useful to work with case vignettes or interview excerpts. For instance, the attribution processes in Kai's descriptions inherently refer to attributions of (dis)ability that are “ascribed as ‘not normal,’ as ‘not capable,’ or as not or only partially capable” (22). This example underscores the significant influence teachers wield over self-concepts [see (19)]. In this respect, the case can be used with Schierz and Thiele's ideas (23) as a “model case” in the context of casuistic casework by referring to the antinomies in PE teacher behavior from Kai's perspective. The reflective consideration and processing of the case with respect to ableist constructs and the associated concealments [see (24)] facilitates the identification of alternative interpretations to facilitate the identification of “useful answers” for inclusion-sensitive action, among other things.

### 2.2 Analysis

We employed a methodological triangulation in our analysis of the material, with the “reconstruction of narrative identity” serving as the fundamental basis of the study, as outlined by Lucius-Hoene and Deppermann (25). The methodological approach is rooted in the narration analysis pioneered by Schütze (26) and incorporates foundational principles of Discursive Psychology (27) and Grounded Theory (28). A manual analysis comprises several steps. Initially, a narrative-structural, sequential analysis is conducted to identify passages that are particularly “dense,” defined as those with a pronounced performative structure, restaging of key narratives, negotiation processes, or even self-presentations. Subsequently, a detailed analysis is carried out at the micro-linguistic level, depending on the research interest. Depending on the content of the discourse and the degree of interest in acquiring knowledge, the following text aspects, for example, are examined in the analysis: agency, categorizations, perspectives, other people's voices, stance indicators and footing, restaging, interviewer orientation, and interactions in the interview. For the main study, the physical positioning of the interviewed individuals in relation to the interviewer was also given consideration. To this end, the model of lived body-(objectified) body-related identity constructions (“Leib-Körper-fundierte Identitätskonstruktion”) according to Gugutzer (29) was used as a methodological extension. This extension is predicated on the assumption that physicality and emotions, operating on an unconscious level, exert a significant influence on the systematically controlled procedure, in both the interview and evaluation phases.^4^

On the one hand, the utilization of the body as an instrument for analysis and investigation enables conscious perception of bodily-affective resonance. Concurrently, this approach facilitates reflection on when one is “touched, repelled, annoyed, turned on, tied up, etc.” (30).^5^ Conversely, this triangulation also offers the opportunity to consider the significance of bodies in their (im)perfection and in their (im)perfection when analyzing narrative interviews. The basis for this triangulation is Dederich's assumption that emotional resonance is particularly important in the context of disability, although it has not played a significant role in the discourse to date. Dederich advocated for “research into feelings and their symbolisation,” as they can provide information about general social “ideas, conventions and practices” (31). This emotional experience plays a role in the interview situation, for example, when body language or paralinguistic elements are changed by emotions.^6^ Dederich sees the physical dialogue that arises in a direct interview situation as an expression of emotions as “exposure to external influences and appeals, which unavoidably precedes any reflexive differentiation between self and other, between self and other” (17). Consequently, this expression of emotions influences both the interviewer and the interviewee and, thus, the entire course of the interview. This point appears to be important for evaluation and interpretation. As a result, the interview, understood as a social practice (32), transcends the confines of the linguistic transcript. In their study, Breuer, Muckel, and Dieris (33) posited that personal affinities can serve as a means to focus attention on emergent phenomena during the research process.^7^ This approach unveiled a novel domain that conceals and harbors “sensitising concepts about the object” (34). This approach has the potential to illuminate the “black box” (34) in qualitative interpretation methods by addressing subjectivity and potential influences on interview progression. This potential is particularly significant given that the interviews were conducted in person, thereby facilitating a physical encounter in which a dysfunctional part of the body—the eyes—was made the subject of discussion. In this methodological approach, we saw a valuable opportunity to recognize and address potential limitations in our own research, including inclusivity.^8^

The evaluation process drew upon the “dignity of the individual case” (35), recognizing the case as a source of knowledge, thereby facilitating an understanding of subjective theories. These theories are shaped by self-images and world views, which are in turn influenced by factors such as self-perception, cultural influences, and historical context [cf. (36)]. For the following analysis, we selected interview sequences on Kai's experiences in PE and extracurricular sports.^9^ We focused our evaluation on the extent to which speeches by professionals shaped the teenager's narratives as a (non)capable actor or on the extent to which normative attributions were adopted and what effects they had. Furthermore, we analyzed the facets of psychological resistance that emerged in the narratives and the extent to which factors for resilience were effective or counterproductive. This analysis was undertaken against the backdrop of enhancing awareness of the pivotal role that educators play in fostering inclusive education, thereby facilitating effective participation.^10^

## 3 Results

Kai, a 15-year-old student, was selected for the following presentation because she had attended a regular school for a longer period of time than the other interviewees, that is, for more than 10 years. In addition, the topic of sports played a central role in her interview.

In her interview, we reconstructed her ambivalence between a strong self-claim and self-doubt that emerged where she tended to have internalized the ascription of her lack of abilities. In the following discussion, we first focus on Kai's recreational sporting activities, specifically football (3.1), followed by an examination of her experiences in both exclusive and inclusive PE (3.2). The following excerpt illustrates our analysis:

### 3.1 More than a hobby: football

In response to the question about her hobbies, which was posed before the interview's narrative segment (01:50–01:54), Kai articulated her enthusiasm for football with a light-hearted laugh: “*I definitely play football*.” Initially, this response elicited surprise from the interviewer (me), yet it simultaneously prompted a heightened awareness of my own preconceived notions of ability. Consequently, I made a conscious effort to distance myself from these assumptions in the subsequent discourse. It is noteworthy that Kai did not discuss blind football, a sport where the goalkeeper is the sole sighted player on the pitch and all others wear blindfolds. Her use of the term “*definitely*” underscored the significance and naturalness of playing football in her life. Subsequent sections of the interview further highlighted the profound significance of conventional football in Kai's self-image as a typical, non-impaired individual:

Well, I can't imagine playing blind football because I've organized my life in such a way that I don't necessarily need my eyes. […] I live like a sighted person. Without sight. That's why I could only play goalkeeper in blind football. (24:41–25:03)

In this act of speech, Kai changed her posture, adopting a seated position that emphasized her statement with a straight back. This statement, which appeared to be in direct contradiction to her previous assertion, “*Sighted. But without eyesight*,” and Kai's self-presentation as an active individual who organizes her life, revealed a strong desire to perceive herself as a sighted person and to be perceived as such by others. In the interview situation, this statement puzzled me because, on the one hand, it testified to unwavering self-will, but on the other hand, I could not help but think of her description of her visual impairment at the beginning of the interview and I could sense the feat of strength that this behavior entailed for Kai. At the same time, this statement, with its defiant, carefree tone of voice and its *positioning* beyond blind football, illustrated a self-description that emphasized Kai's struggle to accept her impairment, which could be interpreted as an identity-threatening moment and was reinforced by the causal adverb “*that's why,*” which introduced the last statement. In Lucius-Hoene's terms, the excerpt unveiled the discursive activities that are in constant flux, whereby individuals construct and present themselves in interactions through the attributes, characteristics, motives, or roles that render them discursively relevant to themselves (self-positioning). Conversely, they concomitantly and complementarily allocate a position to their interaction partners (external positioning) (37).

To provide support for her argument, Kai then stated that she attended a training session for blind football, although she did not specify the time period. However, they were “*only 30-year-old men and then I was always knocked over and then I didn't want to go there anymore*” (25:23–25:31). Nevertheless, she could certainly imagine taking part in the Paralympics, a statement that could be interpreted as in strong contrast with what was previously said and as providing an indication of her narrative identity work in tension between normal vs. (visually) impaired.^11^ Notwithstanding, Kai stated several times that being “*normal*” despite her impairment was very important to her. Another interpretation could be that Kai perceived the Paralympics are noticeably different from the disabled sports previously described by Kai due to their exclusive, highly competitive nature and that she was therefore more willing to accept the Paralympics as a “real” sport.

In the interview with Kai, the topic of football occupied a significant portion of the discussion. In relation to her friendships during her primary school years, she stated, “*I frequently encountered garbage cans from that perspective.*” (I, the interviewer, laughed). Kai continued, “*However, I engaged in outdoor football activities with them* [these friends, AB/SE]” (06:16–06:26). She made a potentially confusing-sounding statement when she stated that despite running into garbage cans, she was still able to play football, which provided insight into the extent of her visual impairment. Despite the tragedy in her story, Kai's intonation inserted a certain level of comedy into the situation, and I (as the interviewer) had to laugh briefly. At the same time, this use of comedy made it clear how much effort it must take for Kai to take part in these football games and to what extent the joint ball games represented an enormous compensatory effort for her to be able to keep up with her sighted classmates in view of her visual impairment. Apparently, however, overlooking the garbage cans was not a problem for Kai or her fellow players on the playground or a situation that would have led to exclusion because she still played football “*with them,*” as outlined by the conjunction “*but,*” *which* introduced the subordinate clause. This positive, empowering experience of self-efficacy through Kai's participation in social interactions (15) in the sense of personal skills also enabled her to integrate her impairment into the narrative with positive connotations. At the same time, it was noticeable that at this point in the interview, as in many others, Kai emphasized the difference between “*them*” (i.e., the children without disabilities) and her. She verbally articulated this distinction only in this instance; the act of playing together in the scenario under discussion united all the participating children. Consequently, Kai did not permit herself to be impeded and did not appear to be deterred from participating by her peers. In Kai's perception, her adaptation to the normally sighted environment was rewarded through the friendships she developed with unimpaired peers: “*Maybe it was different for other blind people or something, they didn't have any friends or anything, but it actually worked well for me*” (06:33–06:41), although the use of the disjunctive “*actually*” served to temper her assertion, leaving room for the interpretation of occasional challenges concerning friendships in the setting of her primary school education. A parallel reading of these two passages revealed the tension within which Kai operated: On the one hand, she differentiated between “*them,*” the normal people, and herself, the “*others*”; on the other hand, she did not present herself. In her narration, she positioned herself as a natural part of the “*other blind people*,” a concept previously highlighted in her assertion that she lives like a sighted person without sight, a notion that we elaborate upon further in the context of her participation in PE at the special school.

In Kai's discourse on her transition from primary school to an inclusive working regular school, football once again assumed a significant role because she ran into a prior acquaintance at a trial training session in her new place of residence before the new school year began. She recounted, “*And then we knew about each other, that we both play football, and then we somehow got together or something*” (11:55–12:03). Kai expressed this coming together without a specific explanation at this point, as shown by her words “somehow […] or something.” In terms of a perspective of ableism, Kai's narrative can be reconstructed as a movement within a normal matrix inherent to school logic because it is “*normal*” to have friends. This new friendship with an unimpaired classmate that developed through sport has lasted throughout her time at the school there and offered a sense of stability, especially in some situations in the hallway in the fifth and sixth grades, in which she was confronted with “*stupid comments*” (12:14–12-15) from other students*.* “*That's why* [because of the friendship, AB SE] *[…] I somehow don't mind things like that*” (12:09–12:18). This friendship seemed to be helpful for Kai's arrival at the new school, but she talked about first having to find a place in the class because “*I moved here*” (11:24–11:27). She went on to explain that some of the other children already knew each other from primary school or through parents who were friends. Kai added a second characteristic that she also found difficult when starting at the new school: “*Just my eyes*” (11:27–11:29).

This quote revealed something that could be found in several places in the interview: Kai focused on her eyes as a part of her that was detached from the rest of her healthy functioning body and found a reason in them when things did not go the way she wanted or hoped. Another example could be found in her narrative when she was invited to a scouting training session with a national sports organization. At the beginning of the sequence, Kai said that her chance to turn her football hobby into a career “*was ruined because of my eyes*” (22:52–22:58). This formulation revealed her own innocence or passivity. The interpretation of the entire interview led to the conclusion that Kai experienced herself as a victim in this context, which implied active obstruction by others. When asked by the interviewer, Kai went on to talk about an invitation to a trial training session with the same organization:

And then they actually had a really good impression of me. You could tell. But then, unfortunately, there was also a girl who had already played against me. And she knew, because there aren't that many people in football who don't see that well, so she knew that I didn't see well. And for her it was all about progressing. And my place would have become hers. And then she ran to her father and said ‘we know her.’ And then her father went to the coach and said, ‘Look there. The one with the cap, she can't see anything.’ Yes, and then the coach came to me and said that my performance was quite good and all that, but unfortunately it wasn't enough. But you could tell it was because of my eyes. (23:14–24:09)^12^

In this narrative passage, Kai allowed other individuals to express their perspectives. This *re-enactment*, in the sense of the evaluation method, was facilitated by the utilization of indirect speech reproduction. According to Lucius-Hoene and Deppermann (25), this approach enables the interviewee to more effectively identify with the narrator's viewpoint within the interview context. The dramatic climax of the narrative was marked by Kai's switch to the present tense, in which she conveyed her father's derogatory words with the same intonation. Furthermore, the use of the conjunctional adverb “*then*” several times in the passage evoked a high degree of narrative resolution; as a listener, I was given a concrete figurative impression. The pejorative depiction of her own person in the literal speech as “*[t]he one with the cap*” marked Kai as a nameless, anonymous person, so to speak, who was reduced to her cap and her visual impairment. It appeared that Kai had previously recounted this narrative on multiple occasions, effectively coming to terms with it in order to cope with the challenging circumstances, which had a detrimental effect on the otherwise positive and identity-forming sport, by creating a psychological distance. This social experience has had a profound and lasting impact on Kai's life, as evidenced by her physical response during the interview, where she slumped down figuratively and gazed down at the table. In terms of resilience strategies, Kai's behavior could be interpreted as seeking to disassociate her sporting failure from her identity. By contrast, she presented her visual impairment objectively and neutrally, simply stating that she cannot see well. This statement highlights Kai's unique approach to managing her visual impairment, with her eyes being cited, in a manner reminiscent of the opening of the narrative, as a testament to her being accepted as a selector, independent of her sporting performance. The interpretation of the entire narration within this context suggests that Kai perceives herself as powerless and subject to the circumstances she has encountered.

Furthermore, Kai expressed anger toward those who do not conform to her ableist-coded understanding of ability. This “entanglement” in a paradox of resisting assigned ability attributions on the one hand and reproducing them on the other (18) is presented in the following section of the text, in which excerpts from the subject of PE at special schools and inclusive working regular schools are interpreted.

Her affinity for ball sports has enabled Kai to participate in a wide range of regular PE lessons without the need for specific adaptations. However, due to her severe visual impairment, it was not possible for her to actively participate in PE in some disciplines without adaptation (40). Contrary to what Tanure Alves et al. (6) found, her sports teacher enabled Kai to participate in real life, even though he did not coordinate the adaptations with her in advance and therefore did not actively involve her as a person in the adaptation process. However, the adaptations, for example in speedminton, which Kai initially rejected, possibly due to the teacher's lack of involvement in the sense of paternalism on the one hand and the visible segregation within the class on the other, ultimately led to participation and even “fun” (40:27). The PE teacher thus revealed an attitude toward inclusion that sees the participation of all as a matter of course, whereby every person in the class is perceived and valued.

### 3.2 PE at the inclusive school and at the special school

In the subsequent part of the interview, Kai expounded on her impressions of the special school she recently began attending after a period of over nine years in an inclusive working regular school. In this passage, Kai's—normatively influenced—astonishment at the motor insecurity of some people and their physical behavior, such as fidgeting, was so evident that she asked herself the question: “*Hm, are you only visually impaired or are there other things?”* (37:51–37:55). Again, this narration used the words “*are you”* to make it clear that Kai did not feel that she was part of this group, as she described the others as outsiders, and at the same time, a negative view of her classmates could be seen in the verbalization “*only visually impaired or are there other things*” She disliked the physical abnormalities, found this behavior strange, and questioned whether the others were not “*disabled”* in more ways than one. She thus established a difference through physical heterogeneity or special behavior within the group of visually impaired people, from which she simultaneously excluded herself by using “*are you*.” This statement can be interpreted as an expression of her own demarcation between “normal” and “abnormal,” as well as the underlying “able” and “non-able” (22). She elaborated on this distinction further in the subsequent interview segment, wherein Kai recounted an experience from PE at a specialized educational institution:

Any normal class with sighted students would now say, if we have a free period, dodgeball or something. And the class would like to (…) do you know ball over the mat? That's what we played today. (38:25–38:41)

Her indignation at the voluntarily chosen and, in her opinion, “*non-normal*” game was clearly audible in her tone of voice. Her linguistically deft positioning of her class as not “*normal,*” and the generalization of sighted students against this lack of normality reinforced the expressiveness of the content. Her confrontation with “normal” attributions of ability became clear again when she talked about the rest of the sports lesson because, despite the didactic adaptation with the gymnastics ball, she injured her finger, “*even though it wasn't hard. Because it just came up so strangely. Because I couldn't/because I couldn't grip it with my hands. It was really big*” (38:41–38:56). This interview excerpt illustrates the tension that Kai feels: On the one hand, her own aspirations are reflected in the real-life implementation possibilities that she recognizes at the level of the didactic adaptations of PE. However, these ability-oriented didactic adaptations of PE also point to immanent attributions of “able” and “not able” on the part of the PE teachers and are recognized and evaluated as such by Kai, as the following quote illustrates: “*And then we wanted to take a smaller one, which was also twice as big as a normal football, and then other people were b/blubbering about it, so to say*” (38:57–39:04). In the interview situation, I realized how her choice of words confused me in that I perceived it as harsh, and the narrated scene became vivid in my mind for a brief moment. With some distance from the interview, I can interpret Kai's statement more neutrally and empathize with her tension more easily. Initially, Kai positioned herself as part of a capable group that wanted to make the game more challenging, marked by the pronoun “*we*.” However, when this implication was not realized, the linguistic difference reappeared through her negatively connoted choice of words “*b/blubbering about it, seen that way”* in combination with the “*others*.” Kai wants to play “normal” games, she rejects blindness-specific sports and thus the special role ascribed to her by the PE teacher and her classmates as a non-capable player. The severity of her statement alludes to the experience of a “double vulnerability” (41) between the physically perceptible physical injury and her experience of psychological injury. The psychological injury also occurred on two levels: first, in Kai's assessment of the “non-normal” game, and second, through the injury that nevertheless happened to her “of all people” in the adapted game.

She then went beyond physical education to discuss “blind-specific” sports, which hold a particular significance at her institution and represent a boundary between normality and non-normality.

Judo/eh judo, horse riding and swimming and so on, and rowing, these are, I don't know, blind-specific sports. A lot of blind and visually impaired people do them, I think. Especially horse riding and stuff like that. And that's just not the norm, I think. (41:04–41:22)

This generalizing statement once again established what normality means to her, and *that “a lot of blind and visually impaired people”* do not correspond with her image of herself. Concurrently, she presented herself as a self-assured individual, exhibiting unwavering determination, and articulating her discontent with the prevailing circumstances, which were novel to her and which Kai, having attended the special school for a mere three months, distinguished from the inclusive PE she was accustomed to at the inclusive working regular school: “ *[…] that's what bothers me the most, these sports that don't exist here*” (41:23–41:26). Thus, Kai expressed not wanting to experience adapted PE but instead desired “*normal*” PE. Her own aspiration is to be treated “normally” (i.e., like a sighted person) and to take on sporting challenges, as she explained in the interview. To achieve these goals, Kai acknowledged that she occasionally experiences physical discomfort or limitations in her performance during sports, or that she “*sometimes gets hit in the face with a ball*” (39:30–39:34) in inclusive PE due to her delayed reaction time, as she elaborated on at another point.

Another sequence, in which she talked about inclusive PE at her inclusive working regular school, illustrated how difficult it is for her to take on a special role. She discussed her class's participation in a badminton match, but Kai opted to play speedminton with a selected student due to the distinct ball and its trajectory characteristics, as well as enhanced visibility. The playing surface was also simplified. She could not cope well with these adaptations, as the following statement shows: “*But these are all things that I didn't want myself, adapted, but it could never have worked any other way*” (39:56–40:05). Kai's insight could be reconstructed linguistically, despite her struggles with accepting the adaptation, as evidenced by her use of “*but*” twice, her use of the subjunctive to emphasize the non-real other possibility “*could never have worked differently,*” and her words “*didn't want, adapted*.” Finally, Kai commented on the scene—surprisingly for me as the interviewer in this situation, as the previous narrative conveyed a different attitude—as follows: “*And in the end, it was also fun, and so on, but accepting this change myself, having to accept it, is difficult for me*” (40:25–40:33), with which she expressed her tension in dealing with otherness, reinforced by the verbal climax “*accepting, having to accept*.” This passage was particularly pertinent in the context of the interview, as it offered rare insight into Kai's emotional confrontation with her visual impairment and the everyday challenges she encountered in school as a result.

Other experiences in the sense of ableist attributions of ability, such as being sorted out due to her visual impairment during a selection training course (42, 43) seemed to have a lasting effect on her, so that uncertainty and struggle are her constant companions, which tend to override and overwrite her positive experiences, as can be seen in the reconstruction of the entire interview.

## 4 Discussion/conclusion

Below, we use the analyses to inform a detailed discussion of the methodological approach, followed by an examination of the content-related aspects. Subsequently, we categorize the results within the context of resilience and inclusion-sensitive teacher education.

### 4.1 Methodological discussion

Given the emphasis placed on the emotional involvement of the researchers, it can be posited that this project constituted an “open methodological endeavour” (34), which is currently to be understood as experimental. This approach prompted the question of the most suitable form for presenting the results. In the present study, a challenge that arose was how to balance the analytical presentation of case descriptions with the incorporation of personal emotions and affinities during the interview and analysis process without compromising the reader's focus on the case descriptions. This question was particularly pertinent given the study's objective to prioritize the experiences of young people in school. A question that arose was how can young people continue to share their experiences if their own emotions become interwoven with the analysis process? A further challenge pertained to the objective of the study, namely, to determine the intended recipient of the findings. To avoid the pitfalls of subsumption logic, it was imperative to analyze the material meticulously and to subject it to rigorous scrutiny in research workshops. The transcription process also presented challenges, including how to alternate the perspective from the analyst to the interviewee during the transcription. This shift in viewpoint was employed to convey the interviewee's emotional state, which can be a source of sensitivity when reviewing case descriptions.

The approach of understanding the interview not solely as a linguistic interaction but also as a socio-linguistic phenomenon still appears to be fundamentally important. This claim can be linked to the field of critical phenomenology, for example. Dickel observed:

The currently emerging field of critical phenomenology looks at how culture affects the body in diverse and difference-sensitive ways. From this perspective, social constructivism and lived experience are not regarded as contradictory; rather, critical phenomenology is understood as an extension of post-structuralist approaches and not as essentialist. (44)

In the future, it will be necessary to look for suitable ways of presentation in order to do justice to this demanding methodological approach.

### 4.2 Content discussion

The evaluations showed that there are barriers to participation and to Kai's acceptance of adaptive participation in both inclusive and exclusive PE but also in PA. These experiences are to be understood as influential and identity-marking (16) and show that she is stuck between her self-assertion and her desire to be normal.

Kai is hindered in her adolescent identity work. She wants to test herself to find out who she is, but the answer from the outside is always “visually impaired,” so to speak, which makes it difficult or even impossible for her to find her own identity.

The analyses showed the opportunities that PE offers as well as PE's potential but also the risks that lie in teachers' attitudes. The body is a pivotal criterion for (performance) assessment in PE (4). Giese and Meier posited the following argument:

Anyone who does not have this [the normal, fit body, AB & SE], appears “unfit” or does not or only partially correspond to the described phantasms of autonomous, constantly optimisable bodies, is threatened with marginalisation and exclusion. The fit body as an unquestioned norm and the normalities produced by it are also reflected […] in subject-specific didactic concepts in sports education and the associated curricular requirements. (22)

Finally, we discuss the implications for the professional development of teachers in the context of inclusion and resilience, emphasizing the importance of developing a reflexive attitude that is sensitive to paradoxes and ambivalence in inclusive sports education.

The following observations can be made: Kai has friends, and through these social relationships, she feels recognized and integrated into communities, which ultimately strengthens her resilience.

However, when evaluated comparatively, Kai's resilience is less pronounced in the interview than that observed in other interviewees (19). Instead, the interviewer is presented with an individual who is insecure and vulnerable, as evidenced by the numerous queries posed throughout the interview. Furthermore, other adverse environmental factors, such as sporting failures (42), appear to have a persistent impact on her, resulting in a state of perpetual insecurity and struggle that overshadows her positive experiences.

In relation to extra-personal or environmental factors that promote resilience, it is important to consider the challenges faced by individuals with visual impairments, such as Kai, in their adolescent identity development. Kai's desire to explore and understand her identity is hindered by external perceptions of her visual impairment, perceptions that serve as a constant reminder of her limitations. This external validation creates significant obstacles for her in navigating her own identity formation. The notion of resilience as being intricately linked with vulnerability as a universal human condition is not only a rational perspective but also one that is of paramount importance. However, it is crucial to identify the extent to which models of resilience promotion, as articulated in the current discourse of responsibility, do not inherently imply that failures are self-inflicted, as Schulz (45) cautioned. This awareness would necessitate a comprehensive realignment of perspectives within educational institutions when formulating resilience promotion strategies.

### 4.3 Conclusion: a resilience-strengthening role for teachers in inclusion-sensitive teaching

Nittel (46) highlighted the significant contributions of teachers, which are often unacknowledged by the teachers themselves. In their roles as biographical custodians, counselors, and, from Mead's perspective, significant others in everyday life, teachers possess a certain scope for pedagogical interventions that are both positively and negatively relevant to life history.

In the logic of Disability Studies, Windisch (47) sees disability as a “process of interaction between physical, psychological, cultural, social and political aspects—and a process that should essentially be (co-)shaped by people with disabilities,” which can be done indirectly by analyzing their perspectives, for example by means of interview excerpts (48–50).

Engagement with Disability Studies by (prospective) educators, coupled with the adoption of a critical perspective, is conducive to the cultivation of an awareness that interrogates standardization, exclusion, and discrimination practices and barriers. Consequently, professionals can prioritize the promotion of student empowerment and participation, shifting away from an institutionally entrenched deficit-oriented perspective (51).

In the context of professionalization in collaborative teaching, Boger and Brinkmann also referred to different approaches:

The experience of inclusion is not additive or cumulative to something but shifts the ways in which the self and the world are perceived. It also makes a difference whether one speaks of making teachers “competent” for inclusive teaching or of practising a different way of dealing with disability, of changing one's attitude towards one’s own (still) non-disabled self, one’s own vulnerability and one's attitude towards others as well as towards a world that is historically trapped in cultures of exclusion. In contrast to the illusion of sovereignty of “competent” behaviour, we are talking here about allowing oneself to be irritated and changed […]. (52)

With reference to Schierz and Thiele from the perspective of sports didactics (23, with reference to 53), it can be explained why it is so relevant to question one's own attitude with the help of a systematic heuristic for one's own (ableist) ideas of normality: “The professionalised practitioner thus proves to be a natural objective hermeneut with regard to the diagnostic parts of his professional practice, without ever having had to learn anything about this methodology.” According to Boger and Brinkmann (52), aspects such as one's own vulnerability and the only temporary non-disability are just as much at the center as aspects of allowing oneself to be irritated (emotionally and in terms of content) by casuistic work, which can subsequently trigger a possible change in personal attitude.

In line with the “inclusive physical education” model, Tiemann (54)^13^ emphasized the inclusion of students: the needs and requirements of students can be seen as important factors for the acceptance of support in the sense of stigma management (56) and successful participation. Also, in line with Goffman, Kolaschinsky (57) pointed to an ableist challenge: “the perception of ‘disabled roles’ from the perspective of ‘non-disabled people.’” According to Kolaschinsky, the consequence of this perception in interactions is that all the personality traits of a disabled interaction partner are affected by the characteristic that deviates negatively from the usual expectations (57). It is possible that sensitization in this area can be achieved through an examination of Kai's story by enabling a reflective perception and processing of the case from a distance. The “model case” of Kai thus reveals various diversity-sensitive readings that contribute to the expansion of teachers' competence to act, without presenting a “correct” solution, but instead by presenting various “usable” solutions (23).

Kolaschinsky also pointed out that sensitizing teachers to such mechanisms beyond the classroom can also be valuable for the students in question by pointing out the distorted self-image of people with visual impairments due to social, dismissive notions of normality, which may be corrected by careful institutional handling:

Since unrealistic admiration for ‘normal’ performance or expectations of “magical” abilities can be reflected in a false perception of one's own performance, adequate feedback regarding the student's performance is particularly important. Otherwise, there is a danger that, in line with the distorted image of others, one's own social acceptance can now be attributed either to an underwhelming compassionate and helpful response from others or to one’s own overstraining efforts to appear “as normal as possible” or “very special.” (57)

The social aspect is also fundamental to creating successful conditions for the school as a place of empowerment, participation, and self-determination. The interviews all emphasized the central importance of the circle of friends, especially for children/young people without disabilities, which Kai did not take for granted. Understanding diversity as something to be valued and not limiting friendships from the outset through certain characteristics of heterogeneity requires more than just bringing children together in classes (57). Rather, it is about the ability to relate to one's fellow human beings and requires a shared learning process between affected and non-affected people through mutual interaction. Ambiguity tolerance is the ability to tolerate discrepancies between one's own perceptions and the perceptions of others to a certain extent. In a first step, this ability can take place in a casuistic discussion before this experience is transferred to the classroom in a second step. It also seems promising to identify and, ideally, to promote factors that contribute to resilience. Some of the factors that have a positive effect on resilience [self-perception, self-control, self-efficacy, social skills, appropriate stress management, problem-solving skills, self-awareness; (15)] can be consciously addressed by teachers in PE through different teaching settings and tasks, so that not only children with VIs but all children in a class can work on their self-esteem. This approach opens potential from the perspective of recognition theory, as illustrated by Grimminger and Gieß-Stüber and (58) for the relevance of the experience of belonging in PE. By analyzing processes of exclusion, the authors showed that students draw on and reinforce established notions of normality when they form groups. The “sensitivity of sports teachers and the didactic staging seem to play an important role” (58).

In the (inclusive) school context, both teachers and students can benefit from this sensitivity in terms of a mutual experience of self-efficacy. Teachers benefit because they realize that their actions can be effective, empowering, and formative for students, or their actions can result in long-term damage to students, thus having a direct influence on school biographies. In a positive sense, this discussion can initiate change, for example, by promoting participation and empowerment for all through a critical and difference-sensitive view of ableism and thus a different attitude.^14^ As a result, situations of limitation are minimized. In this kind of interaction, students can feel that they are accepted and recognized as individuals and that they have the right to have a say and participate (60) in the design of lessons (e.g., at the level of assistive technologies or other adaptations). Such realizations can strengthen the relationship level and ultimately have a positive impact on the classroom atmosphere—whether in an inclusive working regular school or special school context—through mutual recognition, appreciation, and self-image, and sustainably empower individuals in their identity construction.

## Data Availability

The datasets presented in this article are not readily available because the participants have released the data for use in this study only. Requests to access the datasets should be directed to Anne Bödicker, anne.boedicker@ph-karlsruhe.de.

## Footnotes

^1^For a comprehensive presentation of the study, in which Kai, along with two additional adolescents with VI, Felix and Jannik, recount their educational experiences, refer to Bödicker (17).

^2^The aspects enumerated in Section 2 can be found in a different form in Bödicker and Akbaba (19).

^3^In the present case, there are diverging self- and other-attributions, as Kai described herself as "sighted, but without eyesight."

^4^Over the course of conducting the detailed analysis and interpretation, the results were regularly presented for discussion in qualitative research workshops. These workshops were conducted with the objective of eliminating subsumption logic and establishing consensus regarding the statements.

^5^In line with this methodological approach, we would also like to invite readers to make use of any feelings of irritation as a means of identifying their own afflictions.

^6^In line with Schierz and Thiele (23), we see a similar potential from working with cases for PE teachers (see Chapter 4).

^7^In the analysis, this procedure entailed, for instance, that after the thematic analysis had been completed, the material was reviewed once more, with other passages that had affected us also being incorporated into the analysis. This methodological approach was also employed to elucidate the subjectivity and preconceptions of the researchers.

^8^For the challenges of the chosen methodological approach, see the Discussion.

^9^The following subjects were examined in the main study: school experiences in general, the transition to an educational institution for students with special needs, confrontations with what is considered to be normal, ableist attributions in relation to school performance, the relationship between visual ability and academic achievement, peer group experiences and dealing with visual impairment in the family.

^10^We also interpreted selected interview passages in various scientific community groups. The results we present are the essential points of all these steps and were determined from the analysis of example sequences presented below as well as others. We use the following passages as characteristic examples of the interviews.

^11^The use of terms such as (not) normal and (not) able was based on Kai's choice of words or the Disability Studies literature and ableist discourses.

^12^Regarding the analysis and interpretation of this passage in various university or interdisciplinary interpretation groups, it is interesting to note how much we as scientists/teachers are also caught up in the “great divide” (38), as statements along the lines of “that can't be right” were immediately made. At the same time, such statements mark the scientific/institutional reproduction mechanisms of ableist ability norms, which Buchner et al. (39) pointed out: “In the course of these demarcations [between disabled/not able and able, AB], an explicit knowledge of who is able emerges. This knowledge is the subject of the description of regimes of ableism and manifests itself as scientific knowledge, as a promise and as coercion: with the ‘great divide,’ in which a hierarchy is inscribed in favor of those who appear to fulfil certain body norms, a pull is created toward the normal as ‘the desirable.’”

^13^Tiemann is considered to be one of the first to advance modelling on the basis of integration theories and sport didactic concepts for dealing with diversity (55). At the level of modifications, she developed the *6 + 1 model of adaptive physical education* (54), which is based on the *TREE* and *CHANGE IT* models and includes practical modifications for sports and exercise programs. The model can certainly be criticized for considering only the teacher as the authority who can make adaptations possible or not, thereby revealing a considerable power imbalance or relationship of dependency in this respect that should be made the subject of a critical reflection.

^14^See person-orientation as an anthropological basis for a school that promotes giftedness (59).
